# A low-cost ex vivo simulator for training in endoscopic hemostasis and perforation management

**DOI:** 10.1016/j.vgie.2026.04.048

**Published:** 2026-05-06

**Authors:** Samer Al-Dury, Sinan Sharba, Yahya Al Hammada, Naif Al-Hakmani, Al-Shueili Halima, Rima Taifour

**Affiliations:** 1 School of Public Health and Community Medicine, Institute of Medicine, University of Gothenburg, Gothenburg, Sweden; 2 Endoscopy Unit, Division of Gastroenterology and Hepatology, Muscat Endoscopy Academy, Medical City for Military and Security Services, Muscat, Oman; 3 Sahlgrenska Academy, Institute of Medicine, University of Gothenburg, Gothenburg, Sweden

## Abstract

**Background and Aims:**

High-risk endoscopic interventions (bleeding control and perforation closure) require deliberate, repeated practice, yet structured hands-on training is scarce. We developed a Muscat Endoscopy Academy Gastric Simulator (MEA-GASTROSIM), a compact, reusable ex vivo upper-GI simulator built from low-cost, locally sourced materials, aligned with international recommendations supporting simulation in competency-based endoscopy training.

**Methods:**

The system uses a custom-made stomach-shaped wooden mold on a stable pedestal, lined with a 2-component silicone layer and an aluminum conductive layer, and covered with ex vivo animal stomach tissue. A transparent acrylic cover recreates the confined working field and permits instructor visualization; a lateral access port approximates esophageal entry. Bleeding is simulated by colored-fluid injection and subtissue tubing connected to syringes for pressure-controlled, pulsatile flow. Perforations and resection-type defects are created as tailored tissue defects to practice endoscopic closure.

**Results:**

MEA-GASTROSIM supports repeatable, escalating scenarios of oozing and spurting hemorrhage and clinically relevant defects, enabling practice of lesion identification, device selection, injection, clipping, coagulation, and closure, including suturing with immediate feedback.

**Conclusions:**

MEA-GASTROSIM offers a realistic, affordable platform for progressive skill refinement in hemostasis and perforation management without exposing patients to risk.

## Indication and rationale

Endoscopic management of GI bleeding and iatrogenic perforation is a high-risk, low-frequency skill set that benefits from deliberate practice and structured simulation-based training.^1^^,^^2^ Although simulation is increasingly recognized within competency-based endoscopy education, access to affordable and reusable models for hemostasis and perforation management remains limited.^3^, ^4^, ^5^ Existing ex vivo and commercial simulators provide important training opportunities but may be costly, bulky, or less adaptable to specific therapeutic scenarios.^6^^,^^7^ We designed a Muscat Endoscopy Academy Gastric Simulator (MEA-GASTROSIM) to combine biological tissue realism, compact footprint, external instructor visualization, and support for multiple therapeutic maneuvers within a low-cost training platform.

## Simulator design, set-up, and ex vivo tissue

**Figure 1:**
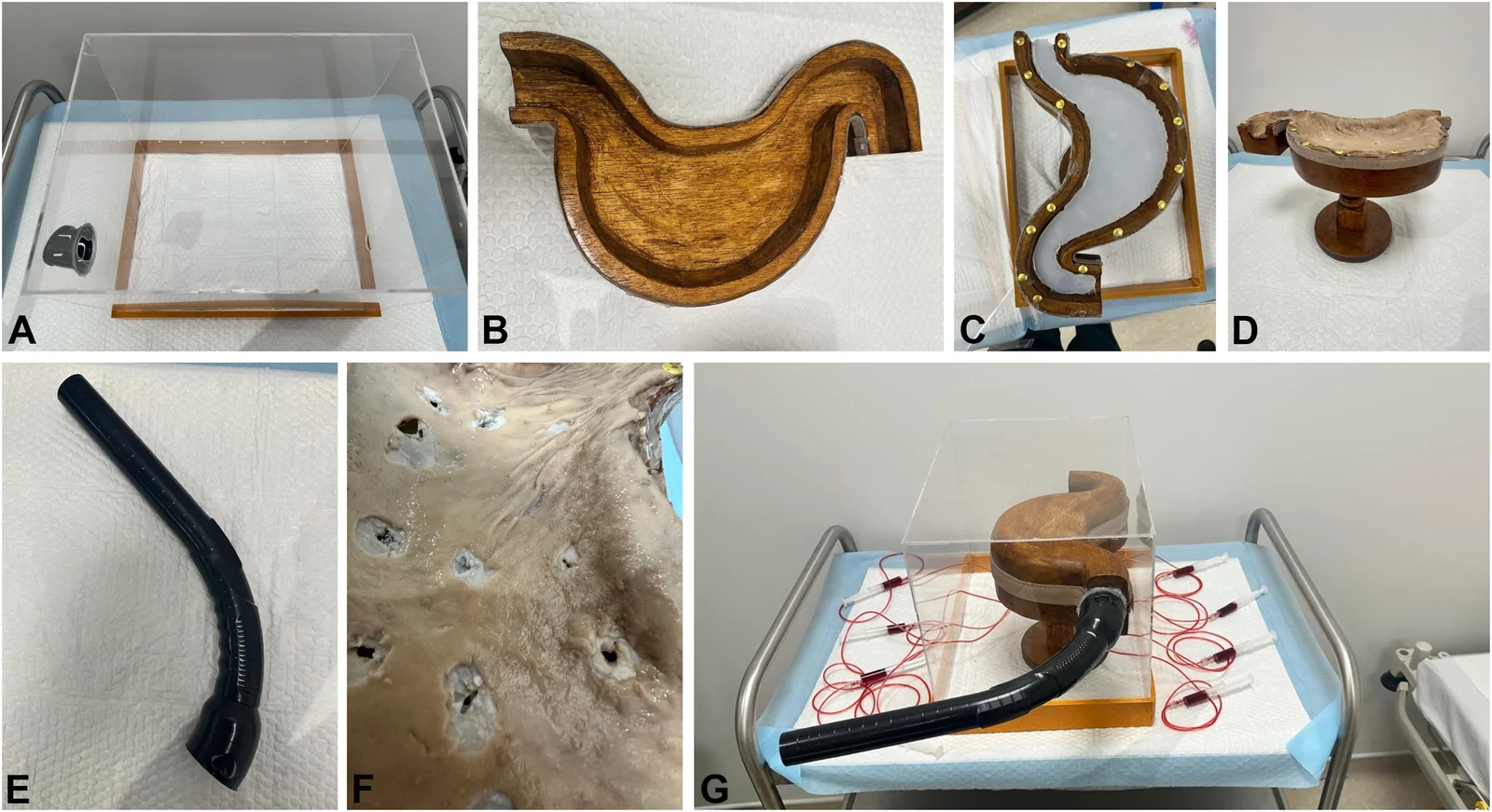
Equipment and mounting of a Muscat Endoscopy Academy Gastric Simulator. **A,** Acrylic enclosure. **B,** Carved wooden stomach mold. **C,** Silicone-lined mold. **D,** Ex vivo stomach mounted on the mold. **E,** Detachable entry tube. **F,** Artificially created tissue defects. **G,** Fully assembled simulator.

MEA-GASTROSIM is built around a hand-carved wooden stomach mold sculpted to reproduce curvature and angulation encountered during upper GI endoscopy (Fig. 1A-G). The mold is mounted on a stable pedestal to standardize scope handling. The wooden component can be produced by a local carpenter using a template, whereas alternative fabrication methods such as 3-dimensional printing may also be feasible. A protective liner is created from a 2-component silicone mixture and applied to the wooden surface to provide a flexible, waterproof interface for tissue fixation. An aluminum layer is then placed above the silicone to provide electrical conductivity and permit use of diathermy during training.

An ex vivo animal stomach is mounted over the layered base and secured circumferentially, providing realistic mucosal and submucosal tactile feedback during injection, clipping, coagulation, and closure maneuvers, including suturing. Tissue can be replaced between sessions at minimal cost. A transparent acrylic (Perspex, Singen, Germany) enclosure is placed over the model to recreate the confined working environment of upper-GI endoscopy while allowing continuous external visualization for instructors. A lateral access port with a detachable flexible tube approximates the esophageal entry angle and facilitates realistic endoscope insertion, torque, and tip deflection (Video 1, available online at www.videogie.org).

## Simulation of bleeding

Bleeding scenarios are generated by 2 complementary methods: (1) injection of colored fluid into/under the ex vivo tissue to simulate oozing or focal intraluminal bleeding and (2) flexible tubing routed beneath the tissue and connected to disposable syringes to deliver pressure-controlled, pulsatile flow to mimic venous or arterial hemorrhage. The model incorporates multiple tubing channels corresponding to different potential bleeding sites. These external channels can be covered so that the trainee does not know in advance which site will be activated. Bleeding severity is adjusted by syringe pressure, tubing diameter, and injection site. Trainees can practice rapid lesion localization, stable targeting, and selection/application of appropriate hemostatic strategies with immediate feedback on effectiveness (Video 1).

## Simulation of perforation

Perforation management is simulated by creating intentional defects in the ex vivo tissue. Defect size and location can be tailored to reproduce iatrogenic perforations encountered during polypectomy/mucosal resection and submucosal dissection. This enables practice of defect assessment, lumen stabilization, device introduction, and endoscopic closure with hemostatic clips or via endoscopic suturing under realistic visual and tactile conditions (Video 1).

## Procedural training and use

A standard endoscope is introduced through the access port and advanced into the lumen. Sessions may follow a structured sequence: scenario briefing, lesion identification and strategy selection, hemostasis or closure, and postprocedural assessment/debriefing. The model supports practice of injection therapy, mechanical hemostasis, coagulation, and defect closure. It may also be used for supervised assessment of lesion targeting, device handling, time to hemostasis, closure quality, and fine tip control.

## Pitfalls, limitations, and troubleshooting

Potential issues include fluid pooling, suboptimal tissue fixation, and loss of tissue elasticity. These are mitigated by sealing the silicone liner and securing the tissue circumferentially. As an ex vivo model, MEA-GASTROSIM does not reproduce peristalsis or respiratory motion. It also does not reproduce loss of insufflation after perforation and therefore cannot fully simulate the deterioration in visualization that may complicate endoscopic closure in clinical practice. In addition, although multiple potential bleeding channels can be externally concealed, bleeding remains externally controlled and does not fully reproduce the unpredictability of real-life scenarios.

## Outcome and educational value

MEA-GASTROSIM is portable, reusable, and reproducible at low cost using widely available materials. It supports progressive skill acquisition in bleeding control and perforation closure, facilitates supervised teaching with instructor visualization, and enables repeated practice without patient risk.

## Disclosure

All authors disclosed no financial relationships.

## Supplementary data

Video 1 Stepwise construction and therapeutic use of a low-cost ex vivo simulator for training in GI bleeding control and perforation management.
